# Efficacy and safety of intravenous nerinetide in acute ischemic stroke patients undergoing endovascular thrombectomy without thrombolysis: a meta-analysis of randomized controlled trials

**DOI:** 10.1007/s00234-025-03847-z

**Published:** 2025-12-16

**Authors:** Abdallah Abbas, Haneen Sabet, Sherief Ghozy, Basant Lashin

**Affiliations:** 1 Faculty of Medicine, Al-Azhar University, Damietta, Egypt; 2 Faculty of Medicine, South Valley University, Qena, Egypt; 3 Department of Neurologic Surgery, Mayo Clinic, Rochester, MN 55902 USA; 4 Department of Radiology, Mayo Clinic, Rochester, MN 55902 USA; 5 Faculty of Medicine, Benha University, Benha, Egypt; 6 Department of Neurologic Surgery, Mayo Clinic, Rochester, MN 55902 USA

**Keywords:** Acute ischemic stroke, Endovascular thrombectomy, Nerinetide, Neuroprotective agent, Intravenous thrombolysis

## Abstract

**Objective:**

This systematic review and meta-analysis aimed to evaluate the safety and efficacy of intravenous (IV) nerinetide in acute ischemic stroke (AIS) patients undergoing endovascular thrombectomy (EVT) without prior or concurrent IV thrombolysis (IVT).

**Methods:**

A systematic search of PubMed, Web of Science, and Scopus was conducted through March 15, 2025, identifying randomized controlled trials (RCTs) comparing EVT plus nerinetide versus EVT plus placebo without IVT. Screening and data extraction were performed independently by two reviewers, with conflicts resolved by a third. Risk of bias was assessed using RoB 2.0. Data were synthesized using RevMan 5.4 with random-effects models, and heterogeneity was evaluated via chi-square and I^2^ statistics.

**Results:**

Three RCTs comprising 726 patients in the IV nerinetide group and 668 in the placebo group were included. Nerinetide did not significantly improve functional outcomes: 90-day modified Rankin Scale (mRS) 0–1 (RR: 1.02, 95% CI: [0.71, 1.47], *P = *0.92) and mRS 0–2 (RR: 1.07, 95% CI: [0.93, 1.22], *P = *0.35). No significant differences were observed in 90-day mortality (RR: 0.89, 95% CI: [0.60, 1.34], *P = *0.59) or adverse events, including symptomatic intracranial hemorrhage (RR: 0.80, 95% CI: [0.44, 1.45], *P = *0.46).

**Conclusion:**

Nerinetide administration during EVT in AIS patients without IVT did not significantly improve functional independence, survival, or safety outcomes compared to placebo. Although preclinical data supported neuroprotection, clinical benefits were not observed, highlighting the challenges in translating neuroprotective strategies into effective stroke therapies.

**Supplementary Information:**

The online version contains supplementary material available at 10.1007/s00234-025-03847-z.

## Introduction

Acute ischemic stroke (AIS) with large vessel occlusion is particularly catastrophic and needs effective reperfusion to be done as early as possible for better clinical outcomes [1]. The intervention of endovascular thrombectomy (EVT) has greatly changed the management of these patients. Even so, a considerable proportion of patients still have poor outcomes despite achieving successful recanalization. Research is being done on nerinetide, a new neuroprotectant targeting postsynaptic density protein-95 (PSD-95), to see if it’s possible to use it as adjunct therapy for reducing ischemic injury in patients between symptom onset and reperfusion [1–3]. Preclinical studies demonstrate nerinetide's capability of reducing infarct size and enhancing neurological recovery [2]. However, its clinical efficacy may be compromised by concurrent thrombolytic therapy: plasminogen activators like alteplase and tenecteplase generate plasmin, which cleaves and inactivates nerinetide, significantly reducing its therapeutic concentrations for several hours after administration [2].

In an effort to remove this unwanted interaction, recent randomized controlled trials (RCTs) have centered on patients with EVT only, with no prior or concurrent intravenous thrombolysis (IVT) [2]. Regardless, some studies have reported incoherent functional outcome, safety, and mortality results. To resolve this ambiguity, we performed a systematic review and a meta-analysis of the RCTs with intravenous (IV) nerinetide versus placebo for AIS patients with EVT and no IVT to clarify the evidence regarding the effect of nerinetide on these patients and to define its therapeutic role.

## Methods

We conducted this meta-analysis according to the Cochrane Handbook for Systematic Reviews of Interventions and Preferred Reporting Items for Systematic reviews and Meta-Analyses (PRISMA) guidelines [4, 5]. We looked through PubMed, Web of Science, and Scopus from the beginning of their records until March 15, 2025, to find all RCTs that compared EVT plus nerinetide to EVT plus placebo in patients with AIS who had not received IVT before or at the same time. If RCTs had a subset of these patients, they were included. The supplementary file (see Supplementary Material 1) contains the search strategy.

Screening was conducted by Rayyan software [6], while data extraction was conducted by Microsoft Excel, each by two independent authors, and a third researcher resolved any disagreements. Data extraction involved data about summary and baseline characteristics: age, country, design, nerinetide dose, sample size, sex, baseline National Institutes of Health Stroke Scale (NIHSS), Alberta Stroke Program Early CT Score (ASPECTS), time from symptom onset to infusion in minutes, and site of occlusion and outcomes: 90-day modified Rankin Scale (mRS), 90-day Barthel index (BI), 90-day mortality, worsening of stroke (defined as progression or hemorrhagic transformation of the index stroke, or increased disability as indicated by a ≥4-point NIHSS change, caused death during hospitalization, or any combination of these), intracranial hemorrhage (ICH), and adverse events (AEs) including any serious AEs (SAEs) and deep venous thrombosis (DVT) or pulmonary embolism (PE). Risk of bias assessment was conducted using the RoB 2.0 tool [7].

We analyzed the data using RevMan version 5.4 [8] using the pooled risk ratio (RR) with a 95% confidence interval (CI), applying the random-effects model. We checked for differences in the data using the chi-square test and I^2^ statistics, considering there was significant variation if the p-value was less than 0.1 or I^2^ was over 50%.

## Results

**Fig. 1:**
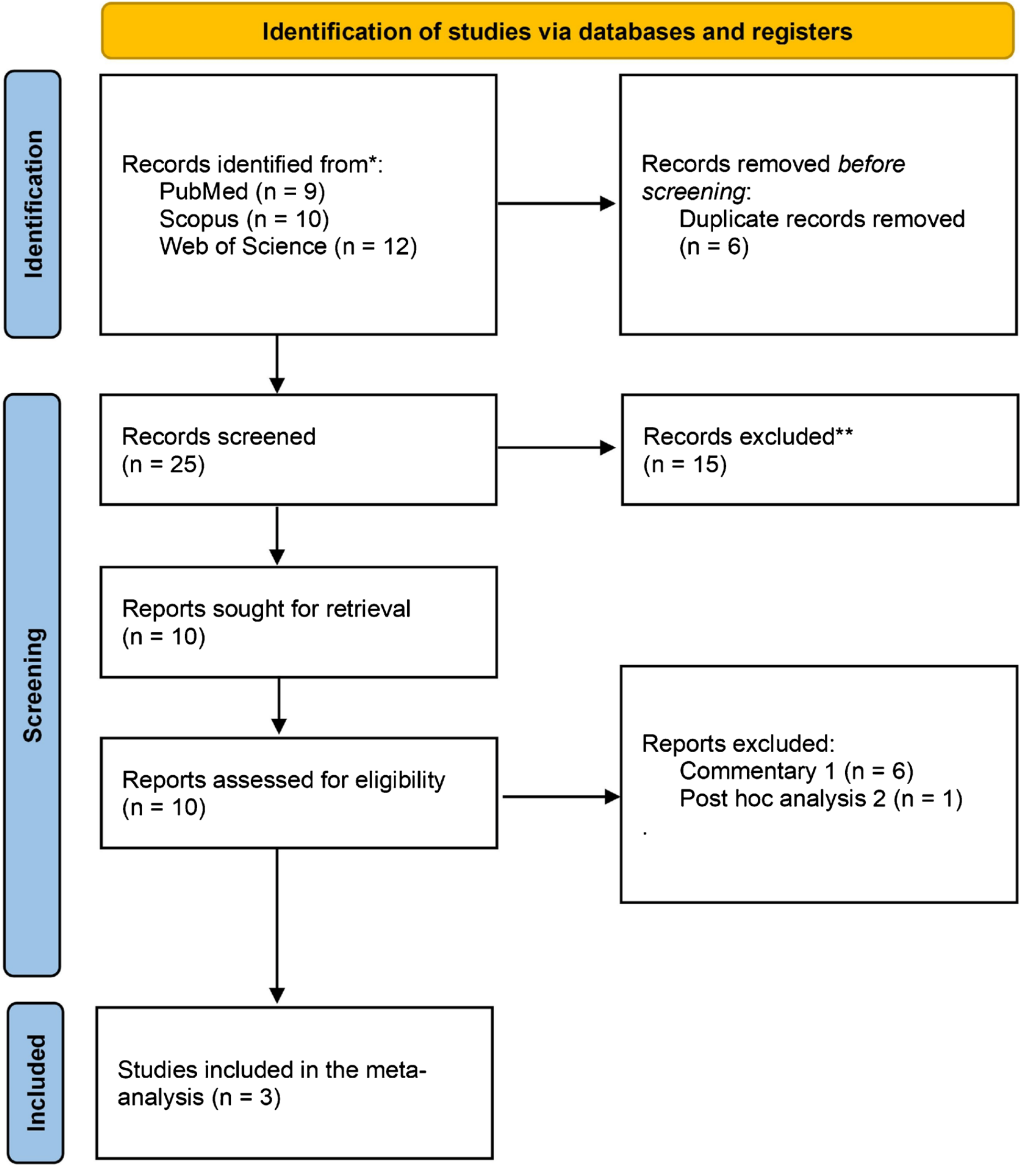
PRISMA flow diagram of study selection. The flowchart illustrates the number of records identified, screened, excluded, and finally included in the meta-analysis. A total of 31 records were initially identified; after removing duplicates and screening titles/abstracts, 10 articles were assessed for eligibility, and 3 randomized controlled trials met the inclusion criteria

We retrieved a total of 31 articles from searched databases, reducing the number to 10 after removing duplicates and conducting title/abstract screening. Ultimately, we included three RCTs [1–3] in our analysis (Fig. 1).

Three included studies were analyzed; one study by Hill et al. [2] focused exclusively on patients undergoing EVT without prior or concurrent IVT, while the other two studies included a subset of such patients, which were selectively included in our meta-analysis, as these studies reported outcomes for this subgroup separately. Across all studies, nerinetide administration was IV. The total number of patients who received EVT alone with nerinetide was 726, while those who received EVT alone with placebo were 668. Before applying subset restrictions, the pooled sample size across all studies was 1,257 for nerinetide and 1,205 for placebo. The pooled median age was approximately 74 years in the nerinetide group and 73 years in the placebo group. The sex distribution was balanced in both groups, with the nerinetide group having 656 males and 601 females and the placebo group having 617 males and 588 females. There was notable variation in time from symptom onset to treatment across studies, ranging from approximately a mean of 62.5–65.5 minutes in Christenson et al. [3], to median of 186–188 minutes in Hill et al. (2020), and 244–300 minutes in Hill et al. [2]. Additional baseline characteristics are detailed in Table 1.

**Table 1:** Summary of included studies and baseline characteristics

Study	Country	Design	Dose of nerinetide	Group	Sample Size	Age in years (median (IQR))	Sex (male: female)	Baseline NIHSS, Median (IQR)	ASPECTS, N (%)	Time from symptom onset to initiation of study drug infusion in minutes, Median (IQR)	Site of occlusion, N (%)	EVT + alteplase (IV thrombolysis), N (%)	EVT alone, N (%)
Christenson 2025	Canada	RCT	2.6 mg/kg (max 270 mg), IV	Reperfusion + Nerinetide	254	74 (65–81)	147: 107	12 (5–19)	N/A	Mean: 62.5 min (SD: 31)	N/A	116 (46%)	53 (21%)
				Reperfusion + Placebo	253	75 (64–83)	141: 112	10 (4–18)	N/A	Mean: 65.5 min (SD: 31)	N/A	96 (38%)	45 (18%)
Hill 2025	Multicenter (Canada, USA, Germany, Italy, Netherlands, Norway, Switzerland, Australia, and Singapore)	RCT	2.6 mg/kg (max 270 mg), IV	EVT + Nerinetide	454	76 (66–83)	228: 226	16 (12–20)	8–10 (304, 67%), 5–7 (147, 32%), 0–4 (3, 1%)	244 (154–415)	ICA (109, 24%), Middle cerebral artery (M1) (277,61%), Middle cerebral artery (M2) (66,15%), No occlusion (2, <1%)	0	454 (100%)
				EVT + Placebo	396	75 (65–83)	201: 195	16 (12–20)	8–10 (271, 68%), 5–7 (119, 30%), 0–4 (6, 2%)	300 (163–500)	ICA (76, 19%), Middle cerebral artery (M1) (268, 68%), Middle cerebral artery (M2) (52, 13%), No occlusion (2, <1%)	0	396 (100%)
Hill 2020	Multicenter (Canada, USA, Germany, Australia, South Korea, Sweden, Ireland, UK)	RCT	2.6 mg/kg (max 270 mg), IV	Reperfusion + Nerinetide	549	71.5 (61.1–79.7)	281: 268	17 (12–21)	8–10 (397, 72.3%)	Onset to randomization: 186 (120–309)	ICA (110, 20%)	330 (60.1%)	219 (39.9%)
				Reperfusion + Placebo	556	70.3 (60.4–80.1)	275: 281	17 (13–21)	8–10 (403, 72.5%)	Onset to randomization: 188 (122–311)	ICA (103, 18.5%)	329 (59.2%)	227 (40.83%)

*RCT* Randomized controlled trial; *IV* Intravenous; *NIHSS* National institutes of health stroke scale; *IQR* Interquartile range; *SD* Standard deviation; *ASPECTS* Alberta stroke program early CT score; *ICA* Internal carotid artery; *M1* First segment of the Middle Cerebral Artery; *M2* Second segment of the Middle Cerebral Artery; *EVT* Endovascular thrombectomy

Two studies by Hill had overall some concerns, while Christenson et al. [3] had an overall high risk of bias; refer to Supplementary Material 2 for more information about each score and justification.

**Fig. 2:**
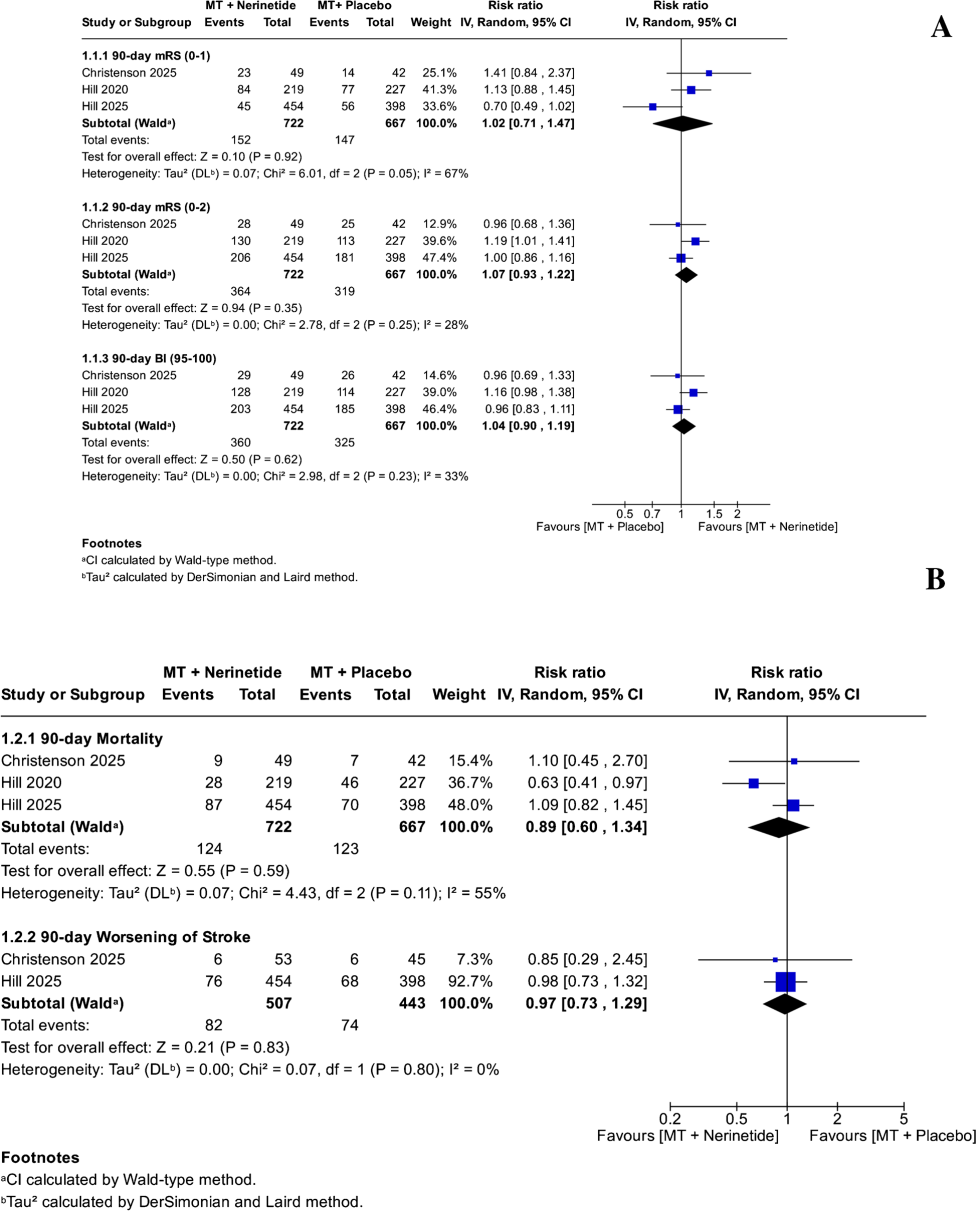
**A** Forest plots of functional outcomes in patients receiving EVT plus nerinetide versus EVT plus placebo. Outcomes include the proportion of patients achieving modified Rankin Scale (mRS) scores of 0–1 and 0–2 and Barthel Index (BI) scores of 95–100 at 90 days post-treatment. Risk ratios (RRs) with 95% confidence intervals (CIs) were calculated using a random-effects model. **B** Forest plots of mortality and worsening of stroke in patients receiving EVT plus nerinetide versus EVT plus placebo. This figure presents the pooled risk ratios (RRs) and 95% confidence intervals (CIs) for 90-day all-cause mortality and 90-day clinical worsening (defined as progression or hemorrhagic transformation of the index stroke, or increased disability as indicated by a ≥4-point NIHSS change, caused death during hospitalization, or any combination of these). Data were analyzed using a random-effects model to assess differences between treatment groups in terms of survival and deterioration following endovascular thrombectomy. MT: Mechanical thrombectomy; mRS: Modified Rankin scale; BI: Barthel index

We did not find a statistically significant difference between using EVT plus nerinetide and EVT plus placebo in the 90-day mRS 0-1 (RR: 1.02, 95% CI: [0.71, 1.47], *P = *0.92, heterogeneity: I^2^ = 67% and *P = *0.05), 90-day mRS 0-2 (RR: 1.07, 95% CI: [0.93, 1.22], *P = *0.35, heterogeneity: I^2^ = 28% and *P = *0.25), and 90-day BI 95-100 (RR: 1.04, 95% CI: [0.90, 1.19], *P = *0.62, heterogeneity: I^2^ = 33% and *P = *0.23) (Fig. 2A). Furthermore, the analysis of 90-day mortality and 90-day worsening of stroke did not reveal a significant difference between the two groups (RR: 0.89, 95% CI: [0.60, 1.34], *P = *0.59, heterogeneity: I^2^ = 55% and *P = *0.11) and (RR: 0.97, 95% CI: [0.73, 1.29], *P = *0.83, heterogeneity: I^2^ = 0% and *P = *0.80), respectively (Figure 2B).

**Fig. 3:**
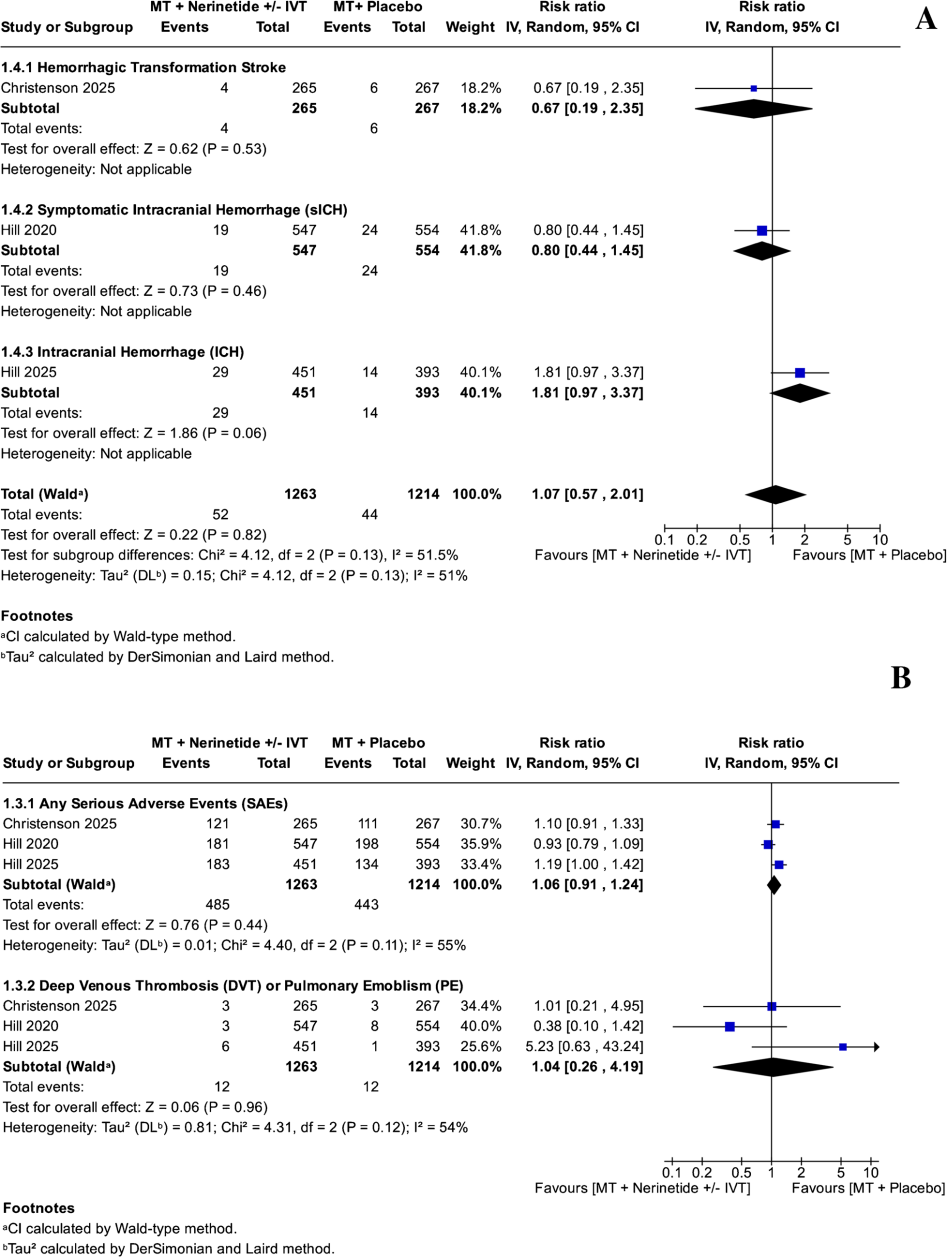
**A** Forest plots of hemorrhagic safety outcomes for EVT plus nerinetide versus EVT plus placebo. Outcomes include hemorrhagic transformation (HT), symptomatic intracranial hemorrhage (sICH), and any intracranial hemorrhage (ICH). Data are presented as pooled risk ratios (RRs) with 95% confidence intervals (CIs) using a random-effects model. **B** Forest plots of adverse events for EVT plus nerinetide versus EVT plus placebo. Outcomes include serious adverse events (SAEs) and thromboembolic events (deep vein thrombosis or pulmonary embolism). Estimates are reported as risk ratios (RRs) with 95% confidence intervals (CIs) under a random-effects model. MT: Mechanical thrombectomy; IVT: Intravenous thrombolysis

We did not find a meaningful difference between using EVT with nerinetide and EVT with a placebo, with or without IVT, in terms of hemorrhagic transformation [HT] (RR: 0.67, 95% CI: [0.19, 2.35], *P = *0.53), symptomatic ICH [sICH] (RR: 0.80, 95% CI: [0.44, 1.45], *P = *0.46), ICH (RR: 1.81, 95% CI: [0.97, 3.37, *P = *0.06]) (Fig. 3A), any SAEs (RR: 1.06, 95% CI: [0.91, 1.24, *P = *0.44, heterogeneity: I^2^ = 55%, *P = *0.11]), and DVT or PE (RR: 1.04, 95% CI: [0.26, 4.19], *P = *0.96, heterogeneity: I^2^ = 54%, *P = *0.12) (Fig. 3B).

## Discussion

In this meta-analysis of three RCTs, it appears that administration of IV nerinetide as an adjunctive IV therapy during EVT in AIS patients without prior or concurrent IVT does not improve functional independence, patient survival rates, or decrease AEs compared to doing EVT alone. Even with the positive preclinical data supporting neuroprotection associated with inhibiting PSD-95, the clinical outcomes only serve to reinforce the persistent difficulty of translating protective strategies into effective therapies for stroke in humans. Additionally, regarding imaging parameters, pre-clinical data have shown promising effects of nerinetide in reducing infarct volume in both transient and permanent stroke models, even when applied up to several hours after injury (>3 hours) [9–11]; however, imaging data were not consistently available in the included RCTs.

Our findings are also in line with the conclusions of the individual trials. Hill et al. [2] specifically focused on EVT patients with no prior exposure to thrombolysis [2]. They found no advantage of using nerinetide in achieving favorable outcomes compared to placebo. Christenson et al. [3] also did not find dominance of nerinetide over placebo in the subgroup of patients undergoing EVT. Regarding Hill et al. [1], they found that patients who received nerinetide without concurrent alteplase achieved better functional outcomes (mRS 0-2) and lower mortality rates. Heterogeneity was low to moderate across most outcomes, suggesting robustness of these neutral findings.

Pre-clinical studies have shown that nerinetide’s neuroprotective effect is time-dependent, with administration within the first 3 hours yielding the greatest benefit [11–13]. Among the included RCTs, Christenson et al. [3] administered nerinetide within 3 hours of symptom onset, before hospital arrival, whereas the Hill et al. trials used a longer treatment window (Hill et al. [2]: median 244 min; Hill et al. [1]: median 186 min). While the primary analysis in the overall suspected stroke population in Christenson et al. [3] was neutral, exploratory analyses showed a signal of benefit in patients with confirmed ischemic stroke who received reperfusion therapy. However, in the context of our meta-analysis focusing on EVT, the specific subgroup of patients in Christenson et al. undergoing EVT (n=98) showed a numerical trend toward benefit (mRS responder: aOR 1.75) that was not statistically significant (95% CI 0.70–4.38). This contrasts with Hill et al. [1], who found that patients who received nerinetide without concurrent alteplase achieved better functional outcomes (mRS 0-2) and lower mortality rates. Overall, these findings suggest that while early administration is promising, the statistical evidence remains insufficient to confirm efficacy in the EVT population specifically.

The rationale for studying nerinetide in EVT-only patients stems from pharmacokinetic concerns: nerinetide is degraded by plasmin generated through tissue plasminogen activators such as alteplase and tenecteplase [2]. Prior studies, including ESCAPE-NA1, showed that nerinetide's efficacy is likely decreased due to the presence of thrombolytics [1]. Unlike the Hill et al. trials [1, 2], Christenson et al. [3] administered nerinetide prior to hospital arrival; consequently, they observed efficacy signals even in patients subsequently treated with IVT, suggesting that prehospital administration may avoid the drug-drug interaction with alteplase previously identified. However, in the other subsidized trials, excluding patients treated with IVT was done to maximize nerinetide’s usage and evaluate the drug's neuroprotective abilities. Even in these optimally designed studies, nerinetide was unable to provide a clinically meaningful benefit. This raises concerning implications as to its proposed mechanism of action, its timing, and that of ischemic injury well beyond molecular pathophysiological mechanisms.

The perspective-neutral results, however, could be due to a number of factors. Patient selection based solely on EVT eligibility may not optimally identify individuals who stand to benefit from neuroprotection. Most modern EVT candidates present with a small core infarct in addition to good collateral circulation, which significantly restricts the amount of salvageable tissue. Nerinetide’s administration time in relation to reperfusion may also not be optimal; despite attempts to rapidly administer nerinetide prior to surgery, the ischemic cascade may have initiated irreversible damage in a multitude of patients. Lastly, stroke pathophysiology includes inflammatory, thrombotic, and excitotoxic processes [14], all of which work heterogeneously that one agent may not be able to intervene single-handedly.

Interestingly, a recent post hoc subgroup analysis of the ESCAPE-NA1 trial further refines the understanding of nerinetide’s therapeutic window by identifying potential effect modification based on baseline ASPECTS scores [15]. In this analysis of 446 patients who did not receive alteplase, nerinetide was associated with a higher rate of favorable 90-day outcomes (mRS 0–2: 59.4% vs. 49.8%), and the treatment effect appeared more pronounced in patients with higher ASPECTS scores [8–10], suggesting greater benefit in those with more preserved brain tissue. Conversely, patients with lower ASPECTS had reduced or no benefit, possibly reflecting irreversible ischemic injury. These exploratory findings, while not definitive, may support the hypothesis that individual tissue viability, as assessed by ASPECTS, may influence nerinetide responsiveness. However, studies included in our analysis also enrolled patients with similarly high ASPECTS values, and the effect was not consistently observed. Thus, further investigation in prospective trials designed to stratify patients accordingly is needed.

Safety issues related to the use of nerinetide, such as ICH, sICH, and SAEs, were not identified in our analysis. The consistent safety profile appears to facilitate additional exploration of neuroprotective maneuvers in acute stroke and comorbid conditions, perhaps with refined criteria for patient selection or multimodal strategies.

Some limitations apply to our study. The small number of included trials diminishes statistical power, precluding meaningful subgroup analyses. Additionally, some concerns characterized two studies, and one study was deemed “high” risk for bias, even though all trials were multicenter and randomized. Because tissue-based imaging endpoints were not consistently reported across included studies, we were not able to analyze those outcomes. Finally, the lack of individual patient data limited more detailed analyses structured around the size of the infarct, collaterals, and time to reperfusion. Given the signal of benefit observed in the FRONTIER trial [3] when nerinetide was administered prior to hospital arrival, thereby bypassing plasmin-mediated degradation, future large-scale RCTs should prioritize pre-hospital administration strategies to definitively test whether ultra-early neuroprotection, combined with standard reperfusion therapies, improves functional outcomes.

In conclusion, this meta-analysis demonstrates that IV nerinetide does not significantly improve clinical outcomes in AIS patients undergoing EVT without IVT. Further work focused on the role of neuroprotection in acute stroke should look to refine intervention timing, increase precision in patient selection, and combine therapies to enhance the success of neuroprotective strategies. Specifically, future large-scale RCTs should prioritize pre-hospital administration strategies to definitively test whether ultra-early neuroprotection, combined with standard reperfusion therapies, improves functional outcomes.

## Supplementary Information

Below is the link to the electronic supplementary material.

Supplementary file1 (DOCX 842 KB)

## Data Availability

Data were publicly available.

## Declarations

### Previous publication

We declare that this study has not been published at all.

### Generative AI in scientific writing

We declare that we have not used AI in writing any part of this abstract.

## References

[1] Hill MD, Goyal M, Menon BK, Nogueira RG, McTaggart RA, Demchuk AM et al (2020) Efficacy and safety of nerinetide for the treatment of acute ischaemic stroke (ESCAPE-NA1): a multicentre, double-blind, randomised controlled trial. The Lancet 395(10227):878–87 10.1016/S0140-6736(20)30258-0 32087818

[2] Hill MD, Goyal M, Demchuk AM, Menon BK, Field TS, Guest WC et al (2025) Efficacy and safety of nerinetide in acute ischaemic stroke in patients undergoing endovascular thrombectomy without previous thrombolysis (ESCAPE-NEXT): a multicentre, double-blind, randomised controlled trial. Lancet 405(10478):560–70 39955119 10.1016/S0140-6736(25)00194-1

[3] Christenson J, Hill MD, Swartz RH, Adams C, Benavente O, Casaubon LK et al (2025) Efficacy and safety of intravenous nerinetide initiated by paramedics in the field for acute cerebral ischaemia within 3 h of symptom onset (FRONTIER): a phase 2, multicentre, randomised, double-blind, placebo-controlled study. The Lancet 405(10478):571–82 10.1016/S0140-6736(25)00193-X 39955120

[4] PRISMA statement [Internet]. [cited 2024 Jul 17]. PRISMA 2020 checklist. Available from: https://www.prisma-statement.org/prisma-2020-checklist

[5] Cochrane Handbook for Systematic Reviews of Interventions [Internet]. [cited 2024 Jul 17]. Available from: https://training.cochrane.org/handbook

[6] Ouzzani M, Hammady H, Fedorowicz Z, Elmagarmid A (2016) Rayyan—a web and mobile app for systematic reviews. Syst Rev. 5(1):210 27919275 10.1186/s13643-016-0384-4 PMC5139140

[7] Sterne JAC, Savović J, Page MJ, Elbers RG, Blencowe NS, Boutron I et al (2019) RoB 2: a revised tool for assessing risk of bias in randomised trials. BMJ 28(366):l4898 10.1136/bmj.l4898 31462531

[8] RevMan Knowledge Base - RMW Knowledge Base [Internet]. [cited 2024 Dec 18]. Available from: https://documentation.cochrane.org/revman-kb/revman-knowledge-base-55377928.html

[9] Bråtane BT, Cui H, Cook DJ, Bouley J, Tymianski M, Fisher M (2011) Neuroprotection by freezing ischemic penumbra evolution without cerebral blood flow augmentation with a postsynaptic density-95 protein inhibitor. Stroke 42(11):3265–70 21903963 10.1161/STROKEAHA.111.618801

[10] Sun HS, Doucette TA, Liu Y, Fang Y, Teves L, Aarts M et al (2008) Effectiveness of PSD95 inhibitors in permanent and transient focal ischemia in the rat. Stroke 39(9):2544–53 18617669 10.1161/STROKEAHA.107.506048

[11] Aarts M, Liu Y, Liu L, Besshoh S, Arundine M, Gurd JW et al (2002) Treatment of ischemic brain damage by perturbing NMDA receptor- PSD-95 protein interactions. Science 298(5594):846–50 12399596 10.1126/science.1072873

[12] Cook DJ, Teves L, Tymianski M (2012) Treatment of stroke with a PSD-95 inhibitor in the gyrencephalic primate brain. Nature 483(7388):213–7 22388811 10.1038/nature10841

[13] Teves LM, Cui H, Tymianski M (2016) Efficacy of the PSD95 inhibitor Tat-NR2B9c in mice requires dose translation between species. J Cereb Blood Flow Metab 36(3):555–61 26661213 10.1177/0271678X15612099 PMC4794097

[14] Deb P, Sharma S, Hassan KM (2010) Pathophysiologic mechanisms of acute ischemic stroke: An overview with emphasis on therapeutic significance beyond thrombolysis. Pathophysiology 17(3):197–218 20074922 10.1016/j.pathophys.2009.12.001

[15] Goyal M, Menon BK, Ospel J, Almekhlafi M, Zerna C, Nogueira R et al (2025) Factors influencing nerinetide effect on clinical outcome in patients without alteplase treatment in the ESCAPE-NA1 trial. J Stroke 27(1):95–101 39916458 10.5853/jos.2024.03139 PMC11834344

